# Perceptions Toward Using Artificial Intelligence and Technology for Asthma Attack Risk Prediction: Qualitative Exploration of Māori Views

**DOI:** 10.2196/59811

**Published:** 2024-10-30

**Authors:** Widana Kankanamge Darsha Jayamini, Farhaan Mirza, Marie-Claire Bidois-Putt, M Asif Naeem, Amy Hai Yan Chan

**Affiliations:** 1 Department of Computer Science School of Engineering, Computer and Mathematical Sciences Auckland University of Technology Auckland New Zealand; 2 Department of Software Engineering Faculty of Computing and Technology University of Kelaniya Kelaniya Sri Lanka; 3 School of Pharmacy Faculty of Medical and Health Sciences University of Auckland Auckland New Zealand; 4 Department of Data Science & Artificial Intelligence National University of Computer and Emerging Sciences (NUCES) Islamabad Pakistan

**Keywords:** asthma risk prediction, artificial intelligence, machine learning, māori perceptions, health system development, mobile phone

## Abstract

**Background:**

Asthma is a significant global health issue, impacting over 500,000 individuals in New Zealand and disproportionately affecting Māori communities in New Zealand, who experience worse asthma symptoms and attacks. Digital technologies, including artificial intelligence (AI) and machine learning (ML) models, are increasingly popular for asthma risk prediction. However, these AI models may underrepresent minority ethnic groups and introduce bias, potentially exacerbating disparities.

**Objective:**

This study aimed to explore the views and perceptions that Māori have toward using AI and ML technologies for asthma self-management, identify key considerations for developing asthma attack risk prediction models, and ensure Māori are represented in ML models without worsening existing health inequities.

**Methods:**

Semistructured interviews were conducted with 20 Māori participants with asthma, 3 male and 17 female, aged 18-76 years. All the interviews were conducted one-on-one, except for 1 interview, which was conducted with 2 participants. Altogether, 10 web-based interviews were conducted, while the rest were kanohi ki te kanohi (face-to-face). A thematic analysis was conducted to identify the themes. Further, sentiment analysis was carried out to identify the sentiments using a pretrained Bidirectional Encoder Representations from Transformers model.

**Results:**

We identified four key themes: (1) concerns about AI use, (2) interest in using technology to support asthma, (3) desired characteristics of AI-based systems, and (4) experience with asthma management and opportunities for technology to improve care. AI was relatively unfamiliar to many participants, and some of them expressed concerns about whether AI technology could be trusted, kanohi ki te kanohi interaction, and inadequate knowledge of AI and technology. These concerns are exacerbated by the Māori experience of colonization. Most of the participants were interested in using technology to support their asthma management, and we gained insights into user preferences regarding computer-based health care applications. Participants discussed their experiences, highlighting problems with health care quality and limited access to resources. They also mentioned the factors that trigger their asthma control level.

**Conclusions:**

The exploration revealed that there is a need for greater information about AI and technology for Māori communities and a need to address trust issues relating to the use of technology. Expectations in relation to computer-based applications for health purposes were expressed. The research outcomes will inform future investigations on AI and technology to enhance the health of people with asthma, in particular those designed for Indigenous populations in New Zealand.

## Introduction

Asthma is a long-term respiratory condition characterized by restricted airflow and rapid fluctuations in airway functioning over a short period [1]. Asthma affects around 262 million people all over the world, and caused 455,000 deaths in 2019 [2]. Hence, optimal asthma management is critical, yet in New Zealand, asthma control continues to be poor, with the rate of asthma exacerbations increasing by a third in the last decade [3]. One of the key issues with asthma management is the lack of awareness of worsening asthma, with exacerbations often being detected too late [4]. Digital technologies and the use of artificial intelligence (AI) can support earlier detection of worsening asthma and improved asthma self-management [5]. There exists an increasing number of digital solutions to support asthma self-management. For instance, the smart peak flow meter used to measure the airflow out of the lungs, and the smart inhaler used for taking medicines, integrated with mobile applications, can help the user automatically record peak flow readings and improve the use of inhaler medications with the audiovisual reminders respectively [6]. There are also digital health management systems and decision support systems that can help control and manage asthma, which have been shown in a recent Cochrane review to significantly improve adherence and asthma control [3,7]. These systems provide opportunities for monitoring treatment behaviors, supporting communication between patients and health care providers, as well as generating data that improves patient-provider interactions [8]. Together, these technologies can assist patients in preventing or mitigating severe outcomes resulting from worsening asthma symptoms. However, despite the vast growth in digital health care solutions to support asthma management, implementation of these technologies into routine practice remains limited [5], and even fewer studies exist to explore how health technologies affect Indigenous populations. One possible reason for poor implementation could be the risk of both consumers and health care providers feeling overwhelmed by the large number of choices and functionalities provided by these technologies [9]. An observational study [10] conducted in Portugal reported that out of 336 participants, only 3% were using an asthma app to monitor asthma and improve inhaler adherence. Another reason is the concerns about data privacy and security [5]. Concerns about data accuracy, training the users, and integration with current patient management systems can limit these implementations [11]. Further, poor eHealth literacy, cost-effectiveness, limitations with technical infrastructure, and gaps in policy and regulatory systems can be causes for these limitations [11]. Understanding the factors that can improve engagement with digital health solutions is key if we are to design and develop solutions to facilitate asthma management as part of routine care.

Recently, there has been an increase in the use of AI and machine learning (ML) techniques for developing disease prediction models embedded in digital health solutions. AI is an advanced computer-based technology used to generate intelligent machines that can perform tasks requiring human intelligence. It allows development of algorithms capable of reasoning, learning, and making data-based decisions. ML is a subset of AI that enables machines to learn and improve from experience without being explicitly programmed. It provides algorithms and techniques that allow computers to analyze and interpret complex patterns and relationships in data automatically. ML algorithms can learn from historical data, identify patterns, and make predictions or decisions based on the learnings. Previous works show the potential of ML to predict asthma attacks and improve asthma management [12,13].

However, ML models have raised ethical concerns, as these tools can potentially exacerbate existing health disparities. A study in the United States found that ML algorithms developed based on electronic health records tend to be biased toward poor and minor ethnic communities and those who lack the financial means for regular and ongoing medical treatment [14]. If the information used to train AI algorithms contains biases, the algorithms will uphold these biases, resulting in inaccurate predictions [15]. This is important to address as there is a risk that use of these AI algorithms could worsen inequities. A narrative review reports that AI algorithms trained in limited demographic groups can lead to a lack of generalizability and unintended social bias [16]. Recent work has demonstrated that state-of-the-art clinical prediction models underperform on women, ethnic, and racial minorities due to the higher absolute number of non-Māori versus Māori represented in the population in New Zealand [17]. New Zealand has one of the highest rates of asthma worldwide, with 1 in 7 children (13%) aged 2-14 years (110,000 children) and 1 in 8 adults (12%; 452,000 adults) currently taking asthma medication [18]. Statistics show that asthma has a higher impact on Māori [18], with asthma admission rates of Māori children being twice the rate of Pākehā (non-Māori) children [19]. Our previous study [20] found that Māori females have the highest mean asthma length of hospital stay compared with other ethnic groups in New Zealand. All these findings highlight the significant impact of asthma on the Māori community. Capacity in technology handling, level of education, perception of communities, and cultural sensitivity are other factors that need to be addressed as technology advances, and to ensure that technologies do not worsen inequities [21]. However, there has been limited research in this area exploring the perceptions of Māori, the Indigenous population of New Zealand, toward the use of AI and technology to manage asthma.

The research aimed to answer the research question “What are the Māori perceptions on using AI and technology to predict the risk of asthma attacks?” We explored to identify factors that may need to be considered when developing ML models and systems to predict the risk of asthma attacks in Māori to ensure the models do not exacerbate existing health inequities. The findings can be used to inform the models and systems designed for asthma risk prediction for Māori and identify specific user requirements to inform customization of future interventions.

## Methods

### Overview

A qualitative study design was followed with inductive thematic analysis.

### Participant Recruitment

Māori diagnosed with asthma were recruited through the team’s Māori researcher’s (M-CB-P) networks and through the research team’s networks following a snowball sampling approach. The Māori researcher engaged with the Māori community through advertisements, social media and word of mouth, in line with the Te Ao Māori (Māori worldview) principle of partnerships and relationships where people’s relationships are central to Māori [22]. The potential participants identified through the above approaches were initially provided with an information sheet, which explained the study to inform them what kind of research they are going to be involved in. Contact details were recorded from those who were happy to engage in the study and satisfied the inclusion criteria. Inclusion criteria required participants to identify as being of Māori ethnicity, having a diagnosis of asthma, provide informed consent, and agree to interview recording or note-taking. The aim was to have a minimum sample of 20 participants to ensure data richness and thematic saturation [23].

### Interview Process

The interviews were conducted by the Māori researcher using a semistructured guide (Multimedia Appendix 1). It was culturally appropriate for the Māori researcher to conduct the interviews. This guide was developed with reference to previous research, which explored Māori experiences and perceptions toward antibiotics for upper respiratory tract symptoms [24], and attitudes toward brain health [25]. The interview guide was reviewed by the team, including the Māori researcher who is affiliated with iwi (tribes) Ngāti Awa and Te Arawa. The interview guide consisted of 6 key sections with multiple open-ended questions. In the first section, the interviewer (M-CB-P) gave an introduction to inform the participant about the purpose and importance of the study. The participant was then asked some basic questions to facilitate their entry into the discussion. In the second section, the interviewer asked the participants open-ended questions about their experience with asthma to elicit information without steering the conversation in a potentially biased direction. The open-ended questions in the third section targeted identifying factors that trigger their asthma. This section of the interview helped the researchers to identify any factors specific to Māori patients with asthma that may need to be considered in asthma risk prediction models to ensure Māori needs are represented. The discussion was then directed toward applying technology in asthma prediction, and Māori views and perceptions toward the use of AI and technology. Each interview took 30-60 minutes to complete and no repeat interviews were required.

Participants were given the choice to respond in either English or Te Reo Māori (Māori language), according to their preference. Participants could invite their caregiver or other support person (eg, the primary caregiver involved in the patient’s daily care) to partake in the interview if the carer was deemed more appropriate. This was solely based on the participant’s choice and there were not any mandatory requirements to do so. The interviews were conducted at a location mutually convenient to the participant and the interviewer. Due to the time constraints for participants for travelling, severe weather conditions, sickness, and availability, web-based interviews through videoconferencing were an alternative offered to participants.

### Ethical Considerations

Participants provided written consent before interviews and verbal consent was obtained beforehand for web-based interviews. Most interviews were audio-recorded with the participant’s consent, except for 2 cases where participants opted for the researcher to take notes instead of recording. Participants were assigned 2-digit unique identifiers, and their data were stored on a password-protected server managed by one of the authors' institutions. As approved by the ethics committee, all participants received a NZ $30 (US $18.30) supermarket voucher to acknowledge their time and contributions. The ethical approval for the study was obtained from the Auckland Health Research Ethics Committee, New Zealand (registration number AH25484).

### Data Analysis

The primary researcher used the transcription tool built within Microsoft Word software to transcribe the recorded interviews. These preliminary transcripts were subsequently refined through manual review, involving repeated listening to the recordings for accuracy. The Māori researcher’s input was sought to enhance the transcripts’ credibility, particularly in identifying Māori terms used by the participants. Hence the transcripts were not returned to the participants for comments or correction. The transcripts were then stored anonymously, using a distinct participant identifier.

The final transcript data underwent a careful procedure for analysis and was imported into NVivo (version 12; Lumivero) to facilitate organized examination and analysis. The analysis process encompassed manual coding, where key themes and patterns were identified through attentive content evaluation. The interview data was analyzed using the Braun and Clarke [26] method of inductive thematic analysis [27]. An initial coding framework was developed by the primary researcher (WKDJ), which was then discussed with a senior researcher (AHYC) and the team’s Māori researcher (M-CB-P). The coding tree had three main branches: (1) concerns over AI use, (2) expectations over AI-based systems and (3) experience with the health care systems. Any disagreements were resolved by consensus discussion within the research team. The coding framework was reviewed and refined, then used to code the interview data, with common themes being extracted iteratively.

Further, we executed a sentiment analysis to understand the opinion of the interview participants regarding the use of AI and technology for asthma attack risk prediction. We used a natural language processing model called BERT (Bidirectional Encoder Representations from Transformers) [28]. BERT is designed to learn by looking at the words in a sentence from both directions (left to right and right to left) at the same time. This bidirectional approach enables BERT to capture the intricacies of context more effectively. Consequently, the pretrained BERT representations can be fine-tuned with the addition of just a single output layer, facilitating the development of state-of-the-art models for various tasks such as question answering and language inference, without requiring substantial modifications to task-specific architectures. We used an existing model in this work as there were not enough data samples to fine-tune the model for our own scenario. We used the BERT model downloaded from the Hugging Face website to conduct the analysis [29]. This model is fine-tuned for sentiment analysis on product reviews in 6 languages: English, Dutch, German, French, Spanish, and Italian. Based on those learnings, it predicts the sentiment of a given input as a number of stars (between 1 and 5). The number of stars shows the positivity or the satisfaction level of the input. We used the existing model in this work as there were not enough data samples to fine-tune the model for our scenario. The answers to the interview question, “What do you think about using technology to help manage your asthma?” were extracted for the sentiment analysis. This data was then fed into the model, and the sentiment scores were obtained from 1 to 5, which were labelled as very negative, negative, neutral, positive, and very positive for analysis.

**Figure 1:**
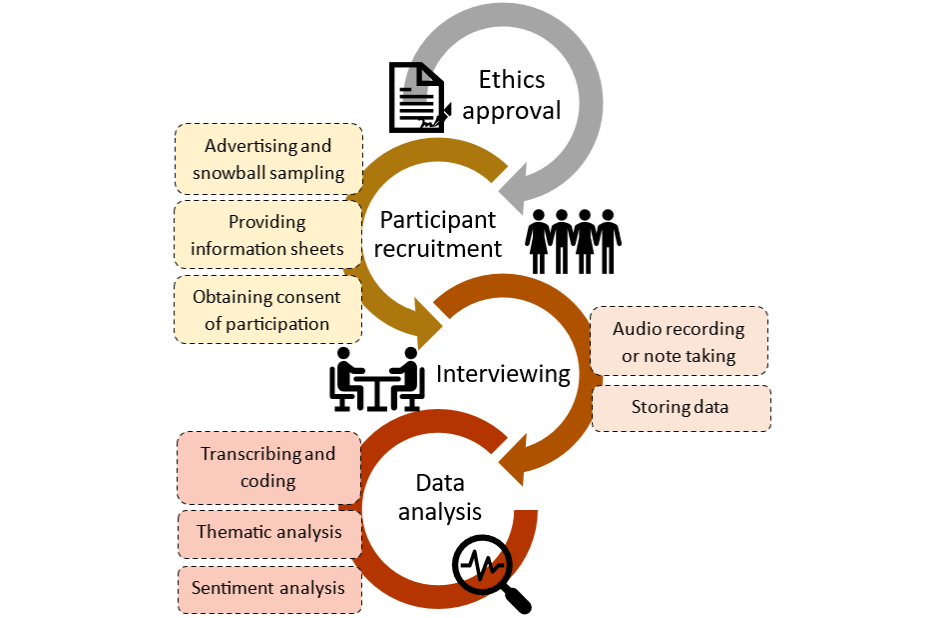
Methodology of exploring Māori perceptions on using artificial intelligence (AI) and technology for asthma risk prediction.

The methodology followed in the study is graphically represented in Figure 1.

## Results

### Participant Characteristics

The final sample consisted of 20 participants (male: n=3, 15%; female: n=17, 85%) aged 18 to 76 (mean 42, SD 16.16) years. None of the participants refused to participate in the interviews after recruitment. The participants came from different regions of the North and South Islands of New Zealand. In total, 2 participants were interviewed together, while the remaining participants were interviewed one-on-one. Out of all participants, 35% had been diagnosed with asthma at less than 5 years of age, while 45% were diagnosed at ages 5-10 years. The remaining participants were diagnosed after the age of 50 years. Most managed their asthma by themselves, while 25% received support from family, friends, or health professionals. Half of the interviews (n=10) were conducted digitally using the Zoom videoconferencing platform, and the rest were conducted kanohi ki te kanohi, according to the participant’s preference.

### Themes

**Figure 2:**
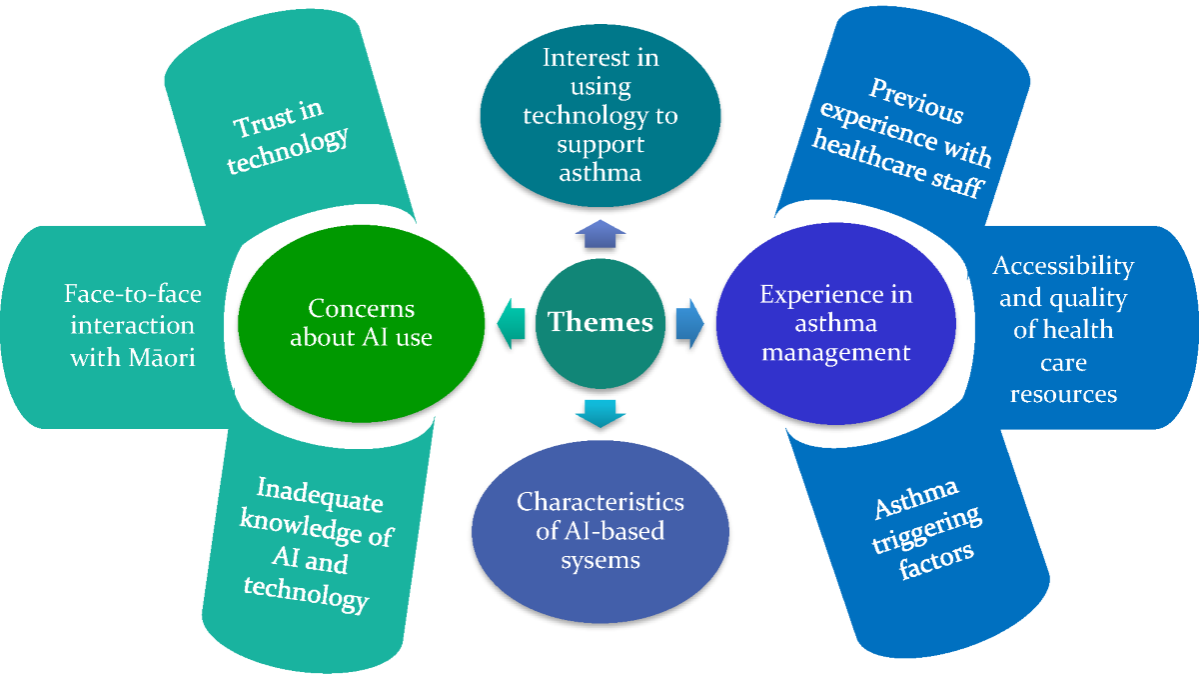
Key qualitative themes relating to the perceptions of Māori towards using AI and technology for asthma attack prediction. AI: artificial intelligence.

In total, four key themes were identified relating to the perceptions of Māori toward the use of AI and technology for the prediction of asthma attacks: (1) concerns about AI use, (2) interest in using technology to support asthma, (3) characteristics of AI-based systems, and (4) experience in asthma management and opportunities for technology to improve care. Themes 1 and 4 were further divided into subthemes, as represented in Figure 2. For Theme 1, concerns about AI use were subdivided into three subthemes: (1) trust about technology, (2) face-to-face interaction with Māori, and (3) inadequate knowledge about AI and technology. Theme 4, experience in asthma management, also consists of three subthemes: (1) previous experience with medical staff, (2) accessibility to health care resources, and (3) asthma-triggering factors. Example quotes for each theme are provided in Multimedia Appendix 2. The following sections present the different themes identified, along with illustrative quotes extracted from the interview data.

#### Theme 1: Concerns About AI Use

##### Trust in Technology

The participants raised several concerns about using AI and technology to predict the risk of asthma attacks. A primary concern was the level of trust that they could place in AI and technology usage. The degree of confidence an individual places in something significantly influences their willingness to embrace and endorse that particular entity or concept [30]. Some participants felt that they could not trust these computer-based systems, particularly as they have received limited information. For Māori, trust is a vitally important aspect of all their relationships. Some participants emphasized that more trust is built up in a relationship with another person of Māori ethnicity.

Like I would be like, yeah, you're going to save me, that would not be something I can trust 100%, ever. … I also believe it can be [obscenity] dangerous when we're relying on that to protect things when everyone's bodies are different because of my health and my journey; I’d be dead if I relied on [obscenity] technology.

E2, F (female), 37 years old

I think trust [emphasizes], there's much trust building up relationships with Māori and stuff like that

E5, F, 38 years old

The researchers note that one way that the general public gets to know AI is through watching movies, where AI is usually portrayed as being used to harm the world. This informal and limited information many people receive makes them believe that AI is bad, and they should not interact with it, especially to manage their health.

…that's what they're thinking of … aww AI is dangerous.

M (male), 76 years old

You know things can harm; you got people talking about AI taking over the world. That's quite scary. AI's not doing what they've been told to do, and I've read so many things about that. And I and I freak out with that.

S7, F, 37 years old

Some participants were hesitant to use AI support to manage their health due to a lack of information about its real benefits. Although they had read about AI, they seemed to have encountered predominantly negative perspectives on its use, which may have contributed to their trust issues.

##### Face-to-Face (Kanohi Ki Te Kanohi) Interaction With Māori

In the context of introducing new AI and technological devices or concepts, some participants preferred engaging in a face-to-face, physical setting with someone from their own communities, for example, a Māori representative. They felt that an individual coming from their own cultural community and possessing cultural insights, would facilitate seamless communication and understanding. Moreover, a few participants felt more comfortable and had greater trust when they received information about these technological applications through the involvement of senior members of the Māori community (kaumatua). Also, a few participants expressed their preference for having a face-to-face option within these technological solutions, “*face-to-face is better*” [M, 23 years old]. This approach would instill a greater sense of ease and comfort in their communication.

My personal thing for Māori would be that kanohi ki te kanohi you know, face-to-face and the fact that they're comfortable with you – A caregiver.

This shows that relationships and culture are important, as having face-to-face interactions with a person from the same culture explaining the information would likely be more readily accepted by the recipient.

##### Inadequate Knowledge of AI and Technology

Interview discussions highlighted that many of the participants wanted to know more about AI and its uses. Several participants candidly expressed that they had limited knowledge of AI and recognized that they wanted to acquaint themselves with these technologies better. In addition, they recognized the importance of broadening their understanding, realizing the positive impact AI could have on their lives and community.

… and when it comes to AI, I don't know much about it. I know little bits.” –

E2, F, 37 years old

Yeah, I think I need to know more about them because I don't know much about technologies and the word AI

S7, F, 37 years old

While the term AI was familiar to them, its practical uses remained unfamiliar. Some participants were apprehensive when the interviewer introduced the concept of using AI to predict asthma attack risks.

Just hearing it to say, you know, to help me with, dealing with my asthma, I just feel nervous about hearing that.

E5, F, 38 years old

…not everybody's confident with technology, and there's been, you know, a lot of rumors around technology and stuff too.

S7, F, 37 years old

The fear of technology stemmed from their limited understanding of AI and related concepts as stated above in reference to AI in movies. Consequently, they tended to accept information given to them at face value due to their lack of information in this area.

#### Theme 2: Interest in Using Technology to Support Asthma

Many participants showed a strong interest in adopting and understanding AI and technology. Some participants already used smart devices such as smartphones and tablets with AI-related features. A few participants were intrigued by the potential of AI to assist in managing their asthma. Most participants responded positively when the interviewer explained how AI and technology can be used to predict asthma attack risk.

Ohh, now you pull up [sic] like that. I think that you know, it's not as scary as I imagined.

E5, F, 38 years old

That sounds awesome, absolutely, yeah, absolutely… It’s just interesting to know. Because, I mean, it’s so much different to manually doing something, like doing it yourself and trying to write a diary on, like, how many times have you done that yet? This and that, and yeah, that'd be interesting.

S6, F, 44 years old

I am a great believer in technology … I think it will benefit humanity immensely. It must be handled the right way.

P15, M, 76 years old

Likewise, many participants generally demonstrated an improved shift in their perceptions of AI, recognizing its potential to make tasks more convenient and expressing confidence in its benefits for humanity when managed correctly.

#### Theme 3: Characteristics of AI- Based Systems

With regard to the attributes of a computer-based system for predicting asthma attack risks, many participants emphasized the importance of utmost simplicity in usage. Notably, they emphasized that the system should be easy to use for older individuals who may encounter challenges when engaging with technology.

When I see my Papa, I see him really struggle to use the tech, so I think they would need to be relatively simple to use. User-friendly. Simple design, simple to use for our elderly.

C4, F, 32 years old

Many participants highlighted that many Māori already need to engage in activities that pose significant complexity, particularly when it comes to interpreting medical terms and navigating the health system. As a result, their preference was for applications characterized by simplicity. While younger participants preferred technologically advanced solutions, they also emphasized the value of straightforwardness, enabling users to navigate the application with minimal effort.

I think if it wasn't too bulky, I guess. We don't have to remember too much.

B9, M, 62 years old

High-tech, Straightforward. Easy to use.

K3, F, 18 years old

When it comes to medical stuff, it needs to be in English, and it needs to be simple, not using scientific words… not using abbreviations, not complicated, no scientific words.

P11, F, 51 years old

Some participants preferred the use of simple English without any scientific words in a proposed system, whereas a few preferred using Te reo Māori, “*An option for Te reo Māori would be great*.” (C4, F, 32 years old). They suggested incorporating Te Reo Māori as an optional selection in technological systems, allowing users to choose based on personal preferences.

#### Theme 4: Experience in Asthma Management and Opportunities for Technology to Improve Care

Some participants discussed their experiences with asthma management under this theme and how these experiences may affect their use of digital technologies to manage asthma. These fell into 3 subthemes relating to accessibility and quality of health care resources, previous experience with health care staff, and triggers for asthma exacerbations.

##### Accessibility and Quality of Health Care Resources

A few participants highlighted the limitations and barriers to accessing health care resources, in particular for those living in rural areas where access to health resources was particularly difficult. Māori living outside urban areas must often travel a long distance to visit a medical clinic to obtain appropriate medical treatments. Some participants described the experience as costly and even life-threatening in an emergency, as arriving at the right place would take longer. One participant mentioned this as follows:

How is it going to help if you are having an attack if you live in the middle of nowhere (farms, the country?).

S1, F, 23 years old

They also highlighted the difference between the quality of the medical services in their region compared to urban areas.

The doctors up here are just so different to Auckland; would I say the DHB (District Health Board) up here is not as good as it is in Auckland, and I noticed that by moving up here. I understand what all my Nana and them we're all talking about now. They're not good here at all.

S6, F, 44 years old

This reflects how some participants are disappointed with health care services in different regions, suggesting that these services do not meet the standards they have experienced or heard about in urban areas.

##### Previous Experience With the Health Care Staff

A few participants were disappointed with the current health care system due to the experiences they had during hospitalizations and treatments. They described general dissatisfaction with the health care system, “*I've been stuck in our medical system until then, so…*” [E2, F, 37 years old]. One participant directly answered the question “Who helps you manage your asthma?” with “It’s me, not our system” [E2, F, 37 years old]*.* A few participants expressed feeling like they had not received proper attention from health care staff, and they expected doctors to listen to them properly and take their concerns seriously.

Yeah, the doctors are great giving out inhalers, but I could do with a review and see if there’s something better on the market. Because you don’t know what’s available. They just kind of put you on something, you think, hey, well, they know, and that’s the best. But it’s not always.

C16, F, 59 years old

I just know that a lot.. and I’m gonna sound racist here, and I don’t mean to be, but a lot of non-Māori therapists don’t work well for Māori.

S17, F, 66 years old

Even though they appreciated the doctor’s service, they discussed how they independently researched different medications themselves, because they felt that the doctor’s prescription was not always correct. Furthermore, they shared that they felt their negative experiences with the health care providers were a result of their ethnicity and a product of a lack of culturally appropriate services.

##### Triggers for Asthma Exacerbations

Almost all the participants did not highlight any specific factors relating to asthma that might be unique to Māori communities or need to be considered in ML prediction models.

Normally it's the shift of like cold to warm, warm to cold it like from one season to the other, like when we're just going in from the end of summer rush into and then entering back at[sic].

E2, F, 37 years old

I feel it only gets worse when I do intense workouts or exercises.

S1, F, 23 years old

...when I start getting sick or anything like that... It like, triggers it. So, if I come down with a cold or anything like that, I get really bad asthma.

S6, F, 44 years old

Dust is another big thing for me. I can't dust my house because I get sick. So someone else has to do it for me, yeah.

S7, F, 37 years old

It was clear that triggers for exacerbations, such as seasonal changes, environmental factors such as pollen and dust, intense workouts and exercises (tiredness), stress, smoking, and other sicknesses were important considerations for them when considering a prediction model.

### Sentiment Analysis

In total, 9 out of 20 (45%) participants had a “neutral” opinion on using AI and technology to predict the risk of asthma attacks, indicating neither positive nor negative thoughts and 4 out of 20 (20%) participants expressed a “very positive” opinion, while 2 out of 20 (10%) participants were “positive.” Altogether, 6 out of 20 (30%) participants showed positivity toward the use of AI and technology for this purpose. However, a quarter of the participants (20%) held a negative viewpoint.

At the time of reporting, we were unable to share the research findings with the participants for their feedback. However, the research team plans to conduct a session where these findings will be shared with both the interview participants and other interested community groups.

## Discussion

### Principal Findings

This is the first study to explore the views, perceptions, and experiences of Māori toward using AI and technology for asthma attack risk prediction, and to explore whether there are any particular factors that should be considered in ML models to predict the risk of asthma attacks. In total, 4 key themes were identified, 3 relating to factors that may affect the engagement and uptake of AI and technology in Māori, and the fourth describing the experience of Māori with the health care system in New Zealand. Participants raised concerns about using AI and technology, with many expressing that they needed more information about AI and technology. This could have been a reason for the majority proportion of the participants being neutral rather than positive or negative about the use of AI and technology to predict the risk of asthma attacks. Historically, Indigenous perspectives—for example the views and perceptions of Māori people—have not had significant influence in guiding the development of emerging technologies such as AI [31]. However, this seems to be changing, with a recent study proposing a method for developing and implementing AI that incorporates mātauranga Māori (Māori knowledge) [32] and government AI policies highlighting the importance of considering impacts of Māori and their perspectives. This will contribute to improving the cultural sensitivity and inclusiveness of AI systems. Providing sufficient information to all users is a key consideration when developing such applications, especially for minority ethnic groups who experience knowledge gaps in technology and limited access to information [33]. Regardless of user age, having sufficient knowledge about the usage of these digital technologies is necessary for potential users to gain the benefits from these technologies to control and manage their disease.

Some questioned the reliance on computer-based predictions and how this may affect the robustness of health care. This highlighted the importance of trustworthiness to participants to engage with computer-based systems. Trust is a measure of the ability to rely on technology, and low trust can lead to disuse of it [30,34], which is a considerable disadvantage, especially as technology could bring about significant benefits for the users. One study [30] identifies the factors affecting trust in technology as characteristics based on the users, the environment, and the technology. This is aligned with this study’s findings that limited knowledge about technology and access to it has created trust issues. Therefore, in the future, computer health app designers and developers should focus on enhancing the reliability for Māori to encourage them use those systems to obtain maximum benefits.

Furthermore, the participants emphasized the importance of face-to-face interactions as part of using technology and devices related to health, in addition to the technology’s introductory sessions. Another study [35] illustrates the significance of incorporating Māori health values such as kanohi ki te kanohi (face-to-face) in perinatal telehealth. This is consistent with the results of our study where participants also highlighted the importance of having a face-to-face element or introduction to technology in order to engage with it. Similarly, another group of researchers investigated the perceptions of using AI in diabetic eye screening [36] and found that the involvement of clinicians in the screening process is a must. Therefore, this face-to-face component is essential in future AI-based systems to make them culturally relevant and applicable.

Although some concerns were expressed about AI and technology usage, participants were open to the idea of using technology to manage their asthma if more information could be provided about the technologies. The percentages of very positive and positive sentiment classes confirm this. A group of researchers explored Māori experiences of telehealth consultations during the COVID-19 lockdown period, and they found how Māori appreciated the support they gained through technology in consulting health professionals [37]. In another study, participants expressed the potential benefits of using mobile health to support their aspirations of hauora (health and well-being) [38].

Participants also suggested some characteristics that technologies and software system developers should consider encouraging uptake and engagement by Māori. The participants’ preference for having Te reo Māori as an option is supported by the finding of other research [39], which reports users having a preference for their own language and highlighting the importance of language for technology uptake. For example, a health professional suggested including language options in digital health systems [37], and that for culturally and linguistically diverse individuals, language can be a barrier to technology use [37,40]. In the New Zealand context, Te reo Māori is culturally important; therefore, it should be considered as an option available in software systems. One study identified the use of Te reo Māori as a symbol of recognition and therefore showing respect to the community within mental health practice [41]. Yet, even though one participant in this study mentioned that they were not fluent in Te Reo Māori, statistical data shows that the use of the Te Reo Māori among Māori has grown from 6.1% in 2018 to 7.9% in 2021 [42]. Hence, following the recommendations from several participants, offering the choice to switch to their native language appears to be a favorable approach for enhancing the usability of these systems among the users. Importantly, this would also be in line with New Zealand's Te Tiriti o Waitangi (Treaty of Waitangi) obligations and encourage wider use of Te Reo within New Zealand society. Te Tiriti o Waitangi is an historical agreement between the British Crown and Māori rangatira (chiefs) and it confirmed among, other things, that Te reo is a taonga (treasure) of Māori culture.

The other feature discussed by the participants, simplicity, was recognized to be important for everyone to minimize the burden of using the system to manage a chronic medical condition such as asthma. During the discussions, participants shared their experiences with the existing health system. One of the concerns they raised was the limited accessibility to health care resources and the quality of it. They questioned the feasibility of travelling from rural to urban areas to receive medical treatment in an emergency. A review paper exploring the experiences of Māori in New Zealand’s public health system has identified practical barriers that have added to the negative experiences of Māori with the health system, such as cost, transport, and time [43]. The paper outlines the reasons for those barriers as financial difficulties, transportation issues, and time allocation difficulties as they need to arrange leave from work or require childcare to attend clinics. Travel time to access care has been cited as a significant barrier to managing health for Māori in other studies particularly those living away from main urban centers [44]. According to Environmental Health Intelligence New Zealand (ehinz), most the Māori population resides in the rural northern and central regions of the North Island [45] where medical services are more limited so access to care in a timely manner is challenging. Technology could address this barrier by supporting web-based consultations, telehealth options and remote monitoring without the need to travel to access in-person consultations.

A few of the participants mentioned the negative experiences they have had in the past when dealing with health care staff during consultations. In addition to apparent and hidden racism and bias, Māori patients and their families believed that the broader aspects of their spiritual and cultural customs were not adequately recognized within the conventional health care system [43]. Technological solutions could improve the experience and interaction issues between Māori and Pākehā by offering more personalized options. These systems could incorporate images or other symbolic elements of Māori culture to enhance user responsiveness, which may not always be feasible in face-to-face interactions. Ensuring that the technology that is developed is culturally responsive and facilitates a more positive experience of the health system is a key consideration going forward.

Furthermore, this study did not identify specific asthma-triggering factors for Māori patients with asthma. This presents an opportunity for AI and technology to analyze data of patients with asthma and determine if there are any specific triggers for Māori individuals experiencing asthma. AI can identify these factors and make predictions based on personalized predictors, leading to more effective and individualized care [12,46,47].

While this study has highlighted important findings, there are several limitations that need to be mentioned. One of the limitations is the small number of participants who participated in the interviews. While we did reach data saturation with 20 participants and the sample of 20 is similar to other qualitative work in this area and may be considered sufficient [41], the current sample of participants may have a gender bias as there were many more females than males. This is important to consider as asthma affects females and males differently and their experiences may therefore be different [48]. Future studies building on this work could explore the influence of sex and gender on asthma and technology experiences and aim to recruit a more balanced sample. The study also conducted a sentiment analysis to present the sentiments of the participants regarding the use of AI and technology to predict the risk of asthma attacks. However, we did not have enough data to fine-tune the model and therefore we used an existing model. In future research a model trained on the same type of data and then used for further analysis would ideally be used. Another limitation of this study is not exploring the impact of Māori culture and knowledge on their experiences of technology. Another limitation of this study is that it did not explore the impact of Māori culture and knowledge on their experiences of technology. Studies involving Māori communities emphasize that “Māori culture at the core of Māori perceptions and experiences” [49], highlighting the need to consider cultural factors [50]. Future research should prioritize examining the role of Māori culture and knowledge to provide a more comprehensive understanding of these experiences. We did not specifically explore access to technology or Digital Divide, asthma assistive technologies, or telehealth, which is another limitation of this study. However, it would be worth differentiating these technologies and exploring them as there can be different opinions about each of them according to personal understandings. Despite this limitation, our findings are still crucial, as this is the first study to investigate this area.

### Conclusions

Digital technologies are increasingly being used to manage health including asthma. With the greater impact of asthma among Māori there is a need to understand their perspectives of digital technology for managing asthma. This study is the first to investigate Māori perspectives on the use of AI and technology for asthma, and to uncover any specific considerations that should be integrated into ML-based asthma attack risk prediction models to mitigate health disparities. Four predominant themes were identified: (1) concerns about AI use, (2) interest in using technology and AI-based systems, (3) characteristics of AI-based systems, and (4) experience in asthma management and opportunities for technology to improve care. Notably, discussions highlighted a need for more information about AI and technology, with concerns about technology trustworthiness. Furthermore, participants’ expectations for a simple, easy-to-use computer-based application, particularly for health care purposes, were identified. These findings hold implications for future research on AI and technology to manage asthma in Māori. It would be worthwhile conducting comparative analysis of other health conditions in Māori. Future work could involve implementing and co-designing technological solutions with Māori Technology has the potential to facilitate equitable health care, and support positive self-management of asthma. Ensuring equitable uptake of technology by considering the perspectives of Māori through all aspects of technology use should be a priority.

## Appendix

Multimedia Appendix 1 Interview guideline.

Multimedia Appendix 2 Example quotes for the themes and subthemes identified in the study.

## Abbreviations

AI: artificial intelligence
BERT: Bidirectional Encoder Representations from Transformers
ML: machine learning

## Data Availability

The datasets generated during and/or analyzed during this study are not publicly available due to participants not consenting to the sharing of data beyond the research team.
