# Supplementary material for: Perceptions Toward Using Artificial Intelligence and Technology for Asthma Attack Risk Prediction: Qualitative Exploration of Māori Views
Source: JMIR Form Res. 2024 Oct 30;8:e59811. doi: 10.2196/59811 (PMC11561449; doi:10.2196/59811)
Supplement: Multimedia Appendix 1 [file formative_v8i1e59811_app1.docx]

## Interview Guidelines

The following open-ended questions were posed to the study participants under each section.

1. Introduction

Introduce the interviewer, study purpose, and rights while going through the information sheet and the consent form.

Asthma attacks affect many people in New Zealand. We are looking at ways to improve asthma care by being able to predict asthma attacks before they happen so that we can keep people well and out of hospital. One way to do this is the use of technology.

The aim of today is to explore your thoughts and feelings about the use of technology in asthma and if there are any specific risk factors for attacks in Māori that we should think about.

Firstly:

- Tell me about you (pepeha)

1. Experience with Asthma

We will start off with talking about your experience with Asthma.

- How do you feel about living with Asthma? How has it affected your life?

For instance: How often do you take medications (such as inhalers)? How often do you go to meet GPs or go to the hospital for asthma?

1. Factors related to Asthma attacks

Let’s talk about the things related to attacks

- What happens when you have an attack?
- What do you do when you have an attack?
- How do you feel?
- What triggers an asthma attack for you?

For instance: Pollen, Temperature, Wind, Pets, Stress

- Have you tried avoiding those triggers, and what have you experienced in doing that?
- Were you able to avoid them, or was there anything that prevents you from doing that?

1. Predicting asthma attacks using technology

One way of predicting asthma attacks is to use technology such as smart devices to monitor asthma (e.g. smart inhalers) and to use artificial intelligence to predict when an attack may happen.

- What do you think about using technology to help manage your asthma?

Are you aware of what AI is – what do you know about AI and its use in healthcare? What do you think of it?

- How would you feel if AI was used to help predict when your next attack might happen?
- What may be the pros/cons for you?
- What factors should be considered when using technology such as AI in Māori living with asthma?
- Why do you think those factors need to be considered?
- Why may it stop you from using technology? What may help?

1. Considerations for Māori when developing risk prediction models

- What factors should be considered when developing risk prediction models for asthma attacks?

e.g. kanohi-ki-te-kanohi, whanaungatanga. How do you think Māori culture and beliefs may be recognised and incorporated when considering risk prediction for asthma attacks?

1. Conclusion

- Is there anything else you would like to tell me that you think would help us in using AI to predict asthma attacks in Māori patients?
- Will I be able to contact you later in case I have additional questions?
