# Supplementary material for: Perceptions Toward Using Artificial Intelligence and Technology for Asthma Attack Risk Prediction: Qualitative Exploration of Māori Views
Source: JMIR Form Res. 2024 Oct 30;8:e59811. doi: 10.2196/59811 (PMC11561449; doi:10.2196/59811)
Supplement: Multimedia Appendix 2 [file formative_v8i1e59811_app2.docx]

Table S1: Example quotes for the themes and sub-themes identified in the study.

| **Sub-theme** | **Example quotes** | **Participant *(Sex, Age)*** |
| --- | --- | --- |
| **Theme 1: Concerns about AI use** | | |
| **Trust in technology** | “… my Papa is dead against it. Papa is dad’s dad, he's really against new technology. Tricknology, you know?” | F, 32 years |
|  | “… I also believe it can be fucking dangerous when we're relying on that to protect things when everyone's bodies are different because of my health and my journey; I’d be dead if I relied on fucking technology.” | F, 37 years |
|  | “… Like I would be like, yeah, you're going to save me, that would not be something I can trust 100%, ever.” | F, 37 years |
|  | “ I think trust [emphasises], there's a lot of trust building up relationships with Māori and stuff like that” | F, 38 years |
|  | “… having something being absorbed by the computer and no physical contact, I think is a bit threatening for them, because where is it all going and what's happening to it?” | A caregiver |
|  | “You know things can harm, you got people talking about AI taking over the world. That's quite scary. AI's not doing what they've been told to do, and I've read so many things about that. And I and I freak out with that.” | F, 37 years |
|  | “… Why are we getting controlled by technology? And who's taking all the data? Who's listening to the technology?” | F, 37 years |
| **Preference for *kanohi ki te kanohi* ( face-to-face) interaction with Māori** | “face-to-face is better” | M, 23 years |
|  | “… for Māori would be that kanohi ki te kanohi, you know, face-to-face and the fact that they're comfortable with you” | A caregiver |
| **Inadequate knowledge of AI and technology** | “… and when it comes to AI, I don't know much about it. I know little bits.” | F, 37 years |
|  | “Yeah, I think I need to know more about them because I don't actually know much about technologies and the word AI” | F, 37 years |
|  | “Just hearing it to say, you know, to help me with, dealing with my asthma, I just feel nervous about hearing that.” | F, 38 years |
|  | “… not everybody's confident with technology, and there's been, you know, there's a lot of rumours around technology and stuff too, so.” | F, 37 years |
| **Theme 2: Interest in using technology to support asthma** | | |
|  | “Ohh, now you pull up like that. I think that, you know, it's not as scary as I imagined.” | F, 38 years |
|  | “That sounds awesome, absolutely, yeah, absolutely… It’s just interesting to know. Because, I mean, it's so much different to manually doing something, like doing it yourself and trying to write a diary on…, that'd be interesting.” | F, 44 years |
|  | “I think it would be learning about it, making sure I'm very well aware of what it's doing, why it's doing what it's doing and making sure there are no side effects to it I guess. Mm. I think that's the main reason why I don't, and I'm not a technology kind of person. Like, I wasn't brought up with technology. So, I've had to adapt to all the technology, learn it all myself…” | F, 37 years |
|  | “I am a great believer in technology” showed the trust that AI can be used to make people’s lives better – “I think it will benefit mankind immensely. It must be handled the right way” | M, 76 years |
| **Theme 3: Desired characteristics of AI-based systems** | | |
|  | “When I see my Papa I see him really struggle to use the tech, so I think they would need to be relatively simple to use. User-friendly. Simple design, simple to use for our elderly.” | F, 32 years |
|  | “I think it’s going to be accessible to all, like young and the old and easy to use basically” | F, 59 years |
|  | “No more than four steps” | F, 66 years |
|  | “I think if it wasn't too bulky, I guess. We don't have to remember too much.” | M, 62 years |
|  | “Make it simple… So, I mean, a lot of people shy away, especially, our people, they shy away from things that are a little bit hard to do so. Maybe if it was a bit easier.” | F, 44 years |
|  | “High-tech, Straightforward. Easy to use.” | F, 18 years |
|  | “When it comes to medical stuff, it needs to be in English, and it needs to be simple, not using scientific words… not using abbreviations, not complicated…” | F, 51 years |
|  | “An option for Te reo Māori would be great.” | F, 32 years |
| **Theme 4: Experience in asthma management and opportunities for technology to improve care** | | |
| **Accessibility and quality of healthcare resources** | “How is it going to help if you are having an attack if you live in the middle of nowhere (farms, the country?).” | F, 23 years |
|  | “The doctors up here are just so different to Auckland; would I say the DHB up here is not as good as it is in Auckland, and I noticed that by moving up here. I understand what all my Nana and them we're all talking about now. They're not good here at all.” | F, 44 years |
| **Prior experience with the healthcare staff** | “It's me, not our system” | F, 37 years |
|  | “I've been stuck in our medical system until then, so…” | F, 37 years |
|  | “Doctors taking our concerns seriously and listening to us” | F, 23 years |
|  | “Yeah, the doctors are great giving out inhalers, but I could do with a review and see if there’s something better out there on the market. Because you don’t really know what’s available. They just kind of put you on something, you think, hey, well, they know, and that’s the best. But it’s not always” | F, 59 years |
|  | “I just know that a lot and I’m gonna sound really racist here and I don’t mean to be, but a lot of non-Māori therapists don’t work well for Māori” | F, 66 years |
| **Triggers for asthma exacerbations** | “Well, if he's been out in the cold and cold and the rain during the day. He will get quite chesty. Or is it the humidity like she said from your shower?” | A caregiver |
|  | “Normally it's the shift of like cold to warm, warm to cold…” | F, 37 years |
|  | “… because I know when I am very active, that really gets me Chesty…” | F, 38 years |
|  | “I feel it only gets worse when I do intense workouts or exercises.” | F, 23 years |
|  | “… when I start getting sick or anything like that… It like triggers it. So, if I come down with a cold or anything like that, I get really bad asthma.” | F, 44 years |
|  | “Dust is another big thing for me.” | F, 37 years |
